# Effects of a Yoga Program Combined with a Mediterranean Diet on Nutritional Status and Functional Capacity in Community-Dwelling Older Adults: A Randomized Controlled Clinical Trial

**DOI:** 10.3390/nu16111601

**Published:** 2024-05-24

**Authors:** María del Carmen Carcelén-Fraile, María Rosalba Martín-Baute, María Isabel Ledesma-Cerrato, Yolanda Castellote-Caballero, Ana María González-Martín, Fidel Hita-Contreras, Javier Cano-Sánchez, Agustín Aibar-Almazán

**Affiliations:** 1 Department of Education and Psychology, Faculty of Social Sciences, University of Atlántico Medio, 35017 Las Palmas de Gran Canaria, Spain; 2 Santa Cruz Rehabilitation Center, 38005 Santa Cruz de Tenerife, Spain; 3 Department of Health Sciences, Faculty of Health Sciences, University of Jaén, 23071 Jaén, Spain; 4 Department of Psychology, Higher Education Center for Teaching and Educational Research, Plaza de San Martín, 4, 28013 Madrid, Spain

**Keywords:** exercise, healthy eating, nutritional state, risk of falls, flexibility, muscular strength, older adults

## Abstract

(1) Background: With the aging population, effective interventions are needed to enhance the health of older adults. This study investigated the combined effects of yoga and the Mediterranean diet on various health outcomes in community-dwelling older adults; (2) Methods: The study employed a randomized controlled trial design with a total of 116 older adults randomized to an experimental group (*n* = 57) that underwent a combined yoga and Mediterranean diet program and a control group (*n* = 59) that did not receive any intervention. Nutritional status was assessed using the Mini Nutritional Assessment, flexibility with the Back Scratch Test and the Chair Sit-and-Reach Test, balance, gait, and fall risk with the Tinetti Scale, and muscle strength with a dynamometer and the 30 s Chair Stand Test; (3) Results: Regarding nutritional status, there were significant differences between the experimental group and the control group (Cohen’s d = 0.02). The participants in the experimental group showed greater balance (11.12 ± 3.01 vs. 10.03 ± 2.35, Cohen’s d = 0.41 and gait (7.63 ± 1.96 vs. 6.69 ± 2.50, Cohen’s d = 0.44) with respect to the control group. In terms of flexibility, the experimental group showed statistically significant improvements in the right arm (Cohen’s d = 0.43), left arm (Cohen’s d = 0.64), right perineum (Cohen’s d = 0.42), and left leg (Cohen’s d = 0.37) Finally, in terms of strength, participants in the experimental group experienced statistically significant improvements in grip strength and lower body strength (Cohen’s d = 0.39 and 0.81, respectively); (4) Conclusions: The study highlights the potential benefits of a 12-week intervention combining yoga with a Mediterranean diet to improve the health and functional capacities of community-dwelling older adults.

## 1. Introduction

Spain has experienced a significant increase in its proportion of older people in recent decades, with the number of people over 65 currently being approximately 20%, a percentage that will continue to increase until the 2060s, which would represent 29% of the total population [1].

The aging process involves a series of changes in various areas, and specifically in the area of nutrition, physiological changes occur that can affect the absorption of nutrients such as vitamin D and iron, as well as alterations in appetite, eating habits, and the risk of malnutrition or undernutrition [2]. Additionally, body composition tends to change in older adults, with an increase in body fat and a decrease in muscle mass [3].

In the physical domain, a decrease in functionality is observed in aspects such as flexibility due to a reduction in physical activity levels, sedentary behavior, and age-related changes, such as modifications in connective tissue [4,5]. Other areas such as balance are also affected in this population, with alterations occurring in this area due to changes in proprioception and muscle tone, which in turn often result in gait changes that can affect dynamic stability and increase the risk of falls [6,7], which can lead to severe bruising, bone fractures, and/or decreased quality of life that are major health concerns for adults older [8]. Similarly, decreases in functionality are observed in older adults due to stiffness and reduced elasticity of tendons or ligaments, which can lead to a decrease in flexibility and the range of free movement in the joints [4,5]. This situation can also cause changes in proprioception and muscle tone, causing alterations in balance and decreases in muscular strength [6,7,8], which can affect the ability to perform daily activities, as well as contribute to loss of independence and decreased quality of life [9,10].

Therefore, it is necessary to implement a series of strategies that contribute to maintaining and/or improving the changes that aging brings with it. Nutrition is key in this aspect [11], as a balanced diet is crucial to supply essential nutrients for the proper functioning of the body [12], and the Mediterranean diet (MD) emerges as a comprehensive approach that can significantly contribute to this global purpose [13]. The updated MD pyramid provides comprehensive details on the essential foods it comprises, such as the consumption of plant-based products (cereals, legumes, nuts, fruits, vegetables, and herbs) and the restriction of red and processed meats. It includes moderate consumption of fish, seafood, eggs, white meats, and dairy products, as well as moderate alcohol consumption, and uses olive oil as the main source of fat. Additionally, clear guidelines are provided on the recommended portions for daily or weekly consumption, which are easy to follow and understand [14].

Physical exercise is essential to maintain good general health at all ages, offering benefits such as improvement of bone and muscle tissues, cardiovascular health, weight control, and improvements in [15,16,17,18]. Among the various forms of exercise for older adults, mind–body therapies stand out for their focus on the connection between nutritional status and physical health [19,20]. Within these therapies, yoga emerges as one of the most beneficial for this specific population, encompassing breathing techniques, physical postures, meditation, and relaxation [21]. Yoga effectively contributes to the assimilation of nutrients thanks to its ability to improve digestion and circulation [22]. The postures and breathing techniques in yoga stimulate the functioning of the digestive organs, promoting more efficient digestion and better absorption of nutrients. This is crucial for overall health, especially in regulating essential metabolic processes in the body [23]. Regarding physical health benefits, yoga is useful for improving range of motion and flexibility thanks to the incorporation of stretches and gentle movements that help maintain or even improve the performance of daily tasks, preventing injuries, and stopping the loss of flexibility and mobility with age [24]. Additionally, many yoga poses require the recruitment and use of muscular strength to maintain the proper position, which contributes to muscle strengthening. This is crucial for maintaining functional independence and preventing the loss of muscle mass associated with aging [25].

Based on the foregoing, the aim of this study is to assess the effects and scope of a combined therapy of yoga along with the Mediterranean diet on the nutritional status and functional capacity of non-institutionalized older adults.

## 2. Materials and Methods

### 2.1. Study Design

A randomized controlled trial was designed to assess the impact of a 12-week yoga intervention on flexibility, balance, grip strength, and lower body strength in non-institutionalized older adults. The study registration was completed prior to its initiation (NCT06304688) and received approval from the Ethics Committee of the University of Jaén (6 March 2024/MAR.PRY).

### 2.2. Participants

**Figure 1:**
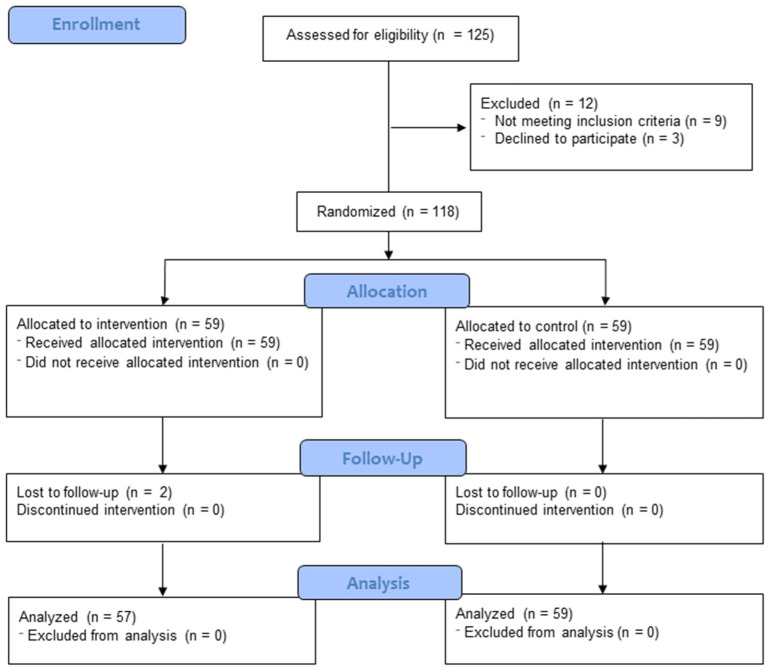
Flow diagram of the study participants.

At the beginning of the study, a total of 125 older adults were contacted, of which 118 met the necessary criteria for inclusion and were therefore enrolled in the study (Figure 1). Participant recruitment took place from October to November of 2023, through phone calls and emails. Those who expressed interest in joining the study completed an informed consent form, in accordance with the Declaration of Helsinki, Good Clinical Practice, and applicable legislation. This form detailed the study’s purposes, methods to be employed, potential risks and benefits, as well as the protection of privacy for collected data. Eligibility criteria for the study included older adults who (i) were 65 years of age or older; (ii) had not participated in yoga programs during the previous 12 months; and (iii) had the ability to understand and execute the instructions and activities proposed in the exercise program. Candidates were excluded if they (i) suffered from any systemic disease (such as neurodegenerative, musculoskeletal, or visual disorders) that would hinder them from carrying out the exercises; (ii) had vestibular diseases or disorders; and (iii) were taking medications affecting the central nervous system, balance, or coordination (such as antidepressants, vestibular sedatives, or anxiolytics).

### 2.3. Randomization

Participant allocation was conducted through a computer-generated random number system, evenly distributing subjects between the experimental group (EG) and the control group (CG) while maintaining a 1:1 ratio. Group assignment was blinded to participants, investigators, and the physiotherapist responsible for the intervention. For this purpose, opaque, sealed envelopes with sequential numbering were used, stored in a secure location accessible only to an individual not directly involved in the research. Once this phase was completed, 59 individuals were placed in the experimental group and another 59 in the control group.

### 2.4. Intervention

Yoga Training. The yoga intervention described in the study was designed to address various aspects of physical fitness comprehensively. Through structured sessions, improvements were promoted in key areas such as strength, flexibility, balance, and stability, which are essential for overall physical health, particularly in older adults. Over a 12-week period, individuals assigned to the yoga group attended a program held twice a week, specifically on Tuesdays and Thursdays, accumulating a total of 24 sessions lasting 60 min each. The sessions were structured into four clearly differentiated parts: (i) starting with 5 min of breathing techniques using Pranayama postures; (ii) followed by 10 min of warm-up exercises focused on joint mobility; (iii) then, 35 min dedicated to the main component, consisting of the execution of various yoga postures; and (iv) ending with 10 min of relaxation techniques including flexibility exercises and stretches. Within the main part of the session, varied postures classified by their position were practiced (Table 1).

Mediterranean Diet. In addition to the yoga intervention, the experimental group received a Mediterranean diet protocol based on the study by Ismail et al. [19] with the following meal plan: (i) carbohydrates constituted 50% of daily intake; (ii) fats represented 35%; and (iii) proteins accounted for 15%. Considering this plan, weekly consumption recommendations were as follows: (i) the use of 320 mL of extra virgin olive oil was recommended; (ii) an intake of 30 g of cereals or bread was advised; (iii) a combination of whole grains, fruits, and vegetables of 125 mL; (iv) consuming 100 g of eggs daily was suggested; (v) legumes and nuts consumed in quantities of 175 mL and 30 g, respectively; (vi) red meat was limited to a weekly portion of 75 g; (vii) a daily consumption of 75 g of fish and poultry was indicated; and (viii) low-fat dairy products were recommended, equivalent to 250 mL of milk, 50 g of cheese, and 175 g of yogurt. Additionally, reducing the intake of certain products such as processed meats, cream, butter, sugary drinks, cookies, bread, and other refined cereals was encouraged.

The members of the control group continued their daily activities unchanged, receiving guidance to promote physical activity while limiting their participation in any type of organized training program, and they were advised to maintain their usual dietary habits.

This helped control for another important variable that could influence the effects of physical activity on the study results. During the 12-week intervention period, regular follow-ups were conducted through periodic telephone calls. This allowed participants to assess their overall physical activity level and monitor any deviations from their usual routines.

### 2.5. Outcomes

All results were collected before and immediately after the end of the intervention period. Prior to allocation, descriptive characteristics such as age, sex, level of education, and marital and occupational status were gathered through self-administered questionnaires in the presence of experienced interviewers. Independent researchers blinded to group allocation performed outcome assessments. Height and weight were assessed using an Asimed T201-T4 adult height scale and a digital precision scale ranging from 100 g to 130 kg (Tefal, Spain), respectively. Body mass index (BMI) was calculated as weight (kg)/height (m^2^) [26].

Adherence to the Mediterranean diet: This was assessed using the 14-item MEDAS questionnaire, validated by the PREDIMED team. This includes 12 questions about the frequency of food consumption and two on common dietary practices in Spain [27]. Each item was scored with zero or one point. A point was awarded for using olive oil as the main culinary fat, preferring white meat over red, and for daily consumption of at least four tablespoons (one tablespoon = 13.5 g) of olive oil (including oil used for frying, dressing salads, etc.), two or more daily servings of vegetables, three or more fruits per day, less than one daily serving of red meats or sausages, less than one daily serving of animal fats, and less than one cup (a cup = 100 mL) of sugary or carbonated drinks. Additionally, a point was granted for consuming at least seven glasses of wine per week, three or more weekly servings of legumes, three or more of fish, a maximum of two industrial bakery products per week, three or more servings of nuts, and at least two servings of sofrito weekly (a sauce made with tomato, garlic, onion, or leeks, cooked in olive oil). The total scoring range varied from 0 to 14 points, with a score of 9 or more considered indicative of adequate adherence to the Mediterranean diet.

Nutritional status: Changes in participants’ nutritional status were assessed using the Mini Nutritional Assessment (MNA), which is a validated tool for detecting and evaluating malnutrition and the risk of malnutrition in older adults (≥65 years). It consists of 18 questions divided into 4 domains: anthropometric assessment (weight, height, BMI, and arm and calf circumferences), global assessment (lifestyle, medication, and mobility), dietary assessment (number of meals and food and liquid intake), and subjective perception of health and nutrition. Each item has a numerical value, and the total score has a maximum value of 30. The MNA classifies nutritional status as adequate (score of 24 to 30), at risk of malnutrition (score of 17 to 23.5), and poor (score less than 17) [28].

Flexibility: To assess functional flexibility, the Back Scratch Test (BST) [29] was used for the upper limbs and the Chair Sit-and-Reach Test (CSRT) [30] for the lower limbs. The BST measured shoulder joint flexibility. It was performed standing, with one hand behind the head descending along the spine, while the other hand was placed on the lower back ascending. The process was repeated alternately. The distance between the tips of the middle fingers was recorded; direct contact equated to “zero”. If there was no contact, the distance was recorded as a negative value (−), and if the fingers overlapped, it was considered a positive value (+). The CSRT was used to assess lower body flexibility, focusing on the hamstring muscles. Participants sat in a chair against a wall for stability and reached to touch their toes with their hands. A direct touch was marked as “zero”. Extensions that did not reach the toes were scored as negative (−), and those that extended beyond were scored as positive (+).

Balance: The Tinetti Scale was used to measure the physical variables of balance, gait, and fall risk [31,32]. This scale is divided into two parts, one measuring static and dynamic balance with 9 items and a maximum score of 17 points and another measuring gait with 7 items, with the highest score of 12 points. The sum of the two parts is used to assess the fall risk. A higher score indicates a lower risk of falling, with 29 being the maximum score achievable. Scores between 19 and 24 points indicate a risk of falls, while scores below 19 indicate low risk.

Muscular Strength: Grip strength was assessed using a hand dynamometer (TKK5001 Grip-A, Takei, Tokyo, Japan), following the method established by the Jamar protocol. Participants stood with an upright posture, shoulders down and in neutral rotation, elbow bent at a 90-degree angle, forearm and wrist in a neutral position. Participants were instructed to exert pressure on a dynamometer, adjusted to the size of their hand so that the fingers formed a right angle at the phalanges. Grip strength was measured for both the right and left hands. These measurements were repeated three times, with a five-minute rest between each measurement, according to the stipulated evaluation procedure [33].

Strength in the lower limbs was assessed in the 30s Chair Stand Test (30s-CST) [34]. Subjects began seated in a chair without armrests, maintaining a straight back and crossing their arms over their chest [35]. They were asked to stand up and sit down as many times as possible within a 30 s period. A greater number of repetitions suggested greater strength in the lower body.

### 2.6. Sample Size Calculation

The sample size calculation for this study is 51 participants per group, which was determined based on the results obtained by Bucht and Donath [36]. Considering the minimum detectable difference between groups, the significance level, and the desired statistical power, we assumed a mean difference of 0.7 units in the strength variable between groups, a significance level of 5% (equivalent to a z-value of 1.96), and a power of 90% (equivalent to a z-value of 1.28) in our scenario. A total of 102 participants were determined, evenly distributed between the control and experimental groups. However, a 15% increase was considered to compensate for potential losses during follow-up, resulting in a total of 118 older adults included in this study, divided between the control group (*n* = 59) and the experimental group (*n* = 59).

### 2.7. Statistical Analysis

All the statistical analyses were performed using IBM SPSS version 25. An exploratory statistical analysis was initially carried out to confirm the minimum and maximum and the presence of missing data. Through the Shapiro–Wilk test (*n* < 50), the normal distribution of the data was confirmed. Next, a univariate analysis was performed, followed by a baseline intergroup analysis to corroborate that there was no significant difference between them (*p* > 0.05) at baseline. In addition, an intragroup analysis was carried out to compare the pre- and post-intervention measures and verify the effect, as well as an analysis of independent groups with ANOVA, which revealed differences between IG and CG. Subsequently, a mixed variance analysis was performed, taking the intervention as the between-group factor while the time of measurement (pre-test and post-test) was the within-subject variable. Finally, a multiple analysis of covariance was performed to adjust for independent variables. A *p* value < 0.05 was considered statistically significant, while the intergroup effect size was calculated using Cohen’s d. An effect size <0.2 was considered insignificant, between 0.2 and 0.5 was considered small, between 0.5 and 0.7 moderate, and >0.8 was considered a large effect size.

## 3. Results

The present study includes 36.96% male participants and 63.04% female participants. Subjects participated in at least 91.6% of the scheduled intervention sessions, and no incidents of injuries or negative reactions were reported during the intervention period (Table 2).

### 3.1. Adherence to the Mediterranean Diet

Regarding adherence to the Mediterranean diet, significant differences appeared in group × time: F(1.114) = 55.325, *p* = 0.000, η^2^ = 0.327, in time: F(1.102) = 107.084, *p* = 0.000, η^2^ = 0.484, and in group: F(1.102) = 72.710, *p* = 0.000, η^2^ = 0.389 (Table 3). The exhaustive analysis of the interaction demonstrates the existence of statistically significant differences between groups in the post-intervention measure, t(114) = 11.729, *p* = 0.000, Cohen’s d = 1.88. Furthermore, the existence of statistically significant differences between the pre- and post-measurement was observed in the group that received the combined yoga training and Mediterranean diet, t(56) = −21.003, *p* = 0.000, Cohen’s d = 2.18.

### 3.2. Nutritional State

According to our findings on nutritional status, significant differences appeared in group × time: F(1.114) = 19.659, *p* = 0.000, η^2^ = 0.147, in time: F(1.114) = 11.641, *p* = 0.001, η^2^ = 0.093, but not in group: F(1.114) = 2.505, *p* = 0.116, η^2^ = 0.021 (Table 2). The exhaustive analysis of the interaction demonstrates the existence of statistically significant differences between groups in the post-intervention measure, t(114) = −2.981, *p* = 0.004, Cohen’s d = 0.05. Furthermore, the existence of statistically significant differences was observed between the pre- and post-measurement in the group that received the combined yoga training and Mediterranean diet, t(56) = −8.820, *p* = 0.000, Cohen’s d = 0.02.

### 3.3. Balance, Gait, and Fall Risk

Regarding balance, significant differences appeared in group × time: F(1.114) = 5.138, *p* = 0.025, η^2^ = 0.043, in time: F(1.114) = 7.704, *p* = 0.006, η^2^ = 0.063, but not in group: F(1.114) = 1.658, *p* = 0.200, η^2^ = 0.014 (Table 2). The exhaustive analysis of the interaction demonstrates the existence of statistically significant differences between groups in the post-intervention measure, t(114) = −2.175, *p* = 0.032, Cohen’s d = 0.40. Furthermore, the existence of statistically significant differences between the pre- and post-measurement was observed in the group that received the combined yoga training and Mediterranean diet, t(56) = −3.116, *p* = 0.003, Cohen’s d = 0.41. With respect to walking, significant differences appeared in group × time: F(1.114) = 14.651, *p* = 0.000, η^2^ = 0.114, but not in time: F(1.114) = 2.869, *p* = 0.093, η^2^ = 0.025, nor in group: F(1.114) = 0.432, *p* = 0.512, η^2^ = 0.004 (Table 2). The exhaustive analysis of the interaction demonstrates the existence of statistically significant differences between groups in the post-intervention measure, t(114) = −2.240, *p* = 0.027, Cohen’s d = 0.42. Furthermore, the existence of statistically significant differences was observed between the pre- and post-measurement in the group that received the combined yoga training and Mediterranean diet, t(56) = −3.595, *p* = 0.001, Cohen’s d = 0.44. Finally, in the Tinetti total score, significant differences appeared in group × time: F(1.114) = 14.163, *p* = 0.000, η^2^ = 0.111, in time: F(1.114) = 8.787, *p* = 0.004, η^2^ = 0.072, but not in group: F(1.114) = 1.769, *p* = 0.186, η^2^ = 0.015 (Table 2). The exhaustive analysis of the interaction demonstrates the existence of statistically significant differences between groups in the post-intervention measure, t(114) = −3.015, *p* = 0.003, Cohen’s d = 0.54. Furthermore, the existence of statistically significant differences was observed between the pre- and post-measurement in the group that received the combined yoga training and Mediterranean diet, t(56) = −4.273, *p* = 0.000, Cohen’s d = 0.54.

### 3.4. Flexibility

Significant improvements were observed in the flexibility of various body parts following yoga treatment/training.

In the flexibility of the right arm, significant differences appeared in group × time: F(1.114) = 10.021, *p* = 0.002, η^2^ = 0.081, in time: F(1.114) = 6.020, *p* = 0.016, η^2^ = 0.050, and in group: F(1.114) = 79.862, *p* = 0.000, η^2^ = 0.412 (Table 2). The exhaustive analysis of the interaction demonstrates the existence of statistically significant differences between groups in the post-intervention measure, t(114) = 2.098, *p* = 0.038, Cohen’s d = 0.43. Furthermore, the existence of statistically significant differences between the pre- and post-measurement was observed in the group that received the combined yoga training and Mediterranean diet, t(56) = 2.306, *p* = 0.025, Cohen’s d = 0.47. In the flexibility of the left arm, significant differences appeared in group × time: F(1.114) = 7.278, *p* = 0.008, η^2^ = 0.060, in group: F(1.114) = 7.013, *p* = 0.009, η^2^ = 0.058, but not in time: F(1.114) = 0.118, *p* = 0.732, η^2^ = 0.001 (Table 2). The exhaustive analysis of the interaction demonstrates the existence of statistically significant differences between groups in the post-intervention measure, t(114) = −3.448, *p* = 0.001, Cohen’s d = 0.64. Furthermore, the existence of statistically significant differences between the pre- and post-measurement was observed in the group that received the combined yoga training and Mediterranean diet, t(56)= −2.071, *p* = 0.043, Cohen’s d = 0.26. In the flexibility of the right leg, significant differences appeared in group × time: F(1.114) = 15.548, *p* = 0.000, η^2^ = 0.120, in time: F(1.114) = 24.338, *p* = 0.000, η^2^ = 0.176, but not in group: F(1.114) = 0.117, *p* = 0.733, η^2^ = 0.001 (Table 2). The exhaustive analysis of the interaction demonstrates the existence of statistically significant differences between groups in the post-intervention measure, t(114) = −2.229, *p* = 0.028, Cohen’s d = 0.42. Furthermore, the existence of statistically significant differences was observed between the pre- and post-measurement in the group that received the combined yoga training and Mediterranean diet, t(56)= −7.542, *p* = 0.000, Cohen’s d = 1.00. In the flexibility of the left leg, significant differences appeared in group × time: F(1.114) = 36.731, *p* = 0.000, η^2^ = 0.244, in time: F(1.114) = 27.469, *p* = 0.000, η^2^ = 0.244, but not in group: F(1.114) = 0.018, *p* = 0.894, η^2^ = 0.000 (Table 2). The exhaustive analysis of the interaction demonstrates the existence of statistically significant differences between groups in the post-intervention measure, t(114) = −1.992, *p* = 0.049, Cohen’s d = 0.37. Furthermore, the existence of statistically significant differences was observed between the pre- and post-measurement in the group that received the combined yoga training and Mediterranean diet, t(56) = 9.414, *p* = 0.000, Cohen’s d = 0.52.

### 3.5. Muscular Strength

Regarding grip strength, significant differences appeared in group × time: F(1.114) = 40.317, *p* = 0.000, η^2^ = 0.261, in time: F(1.114) = 7.149, *p* = 0.009, η^2^ = 0.059, but not in group: F(1.114) = 0.277, *p* = 0.600, η^2^ = 0.002 (Table 2). The exhaustive analysis of the interaction demonstrates the existence of statistically significant differences between groups in the post-intervention measure, t(114) = −2.109, *p* = 0.037, Cohen’s d = 0.39. Furthermore, the existence of statistically significant differences was observed between the pre- and post-measurement in the group that received the combined yoga training and Mediterranean diet, t(56)= −15.994, *p* = 0.000, Cohen’s d = 0.38. Regarding lower body strength, significant differences appeared in group × time: F(1.114) = 18.392, *p* = 0.000, η^2^ = 0.139, in time: F(1.114) = 4.757, *p* = 0.031, η^2^ = 0.040, and in group: F(1.114) = 6.766, *p* = 0.011, η^2^ = 0.056 (Table 2). The exhaustive analysis of the interaction demonstrates the existence of statistically significant differences between groups in the post-intervention measure, t(114) = 4.376, *p* = 0.000, Cohen’s d = 0.81. Furthermore, the existence of statistically significant differences between the pre- and post-measurement was observed in the group that received the combined yoga training and Mediterranean diet, t(56) = 6.908, *p* = 0.000, Cohen’s d = 0.58.

## 4. Discussion

This randomized controlled trial was designed to analyze the effects of a yoga intervention combined with adherence to a Mediterranean diet over 12 weeks on the nutritional status, flexibility, grip strength, balance, gait, fall risk, and lower body strength in non-institutionalized older adults. Yoga was selected as an exercise option in our study due to its accessibility, adaptability, and comprehensive benefits, making it suitable for a wide range of participants, including those who may find conventional strength training too rigorous [37]. Unlike strength training, yoga not only improves flexibility and balance, crucial for fall prevention and mobility enhancement in older adults, but also offers significant benefits in stress reduction and mental health through breathing techniques and meditation [38]. Additionally, yoga contributes to muscular strengthening by utilizing body weight-based postures to enhance endurance and muscle strength more gently compared to weight lifting, making it more viable for individuals with varying physical abilities and health conditions [39]. Following this rationale, the main findings of this study demonstrate significant improvements in nutritional status, balance, gait, fall risk, flexibility, and muscular strength. These results have important clinical implications for this population, not only regarding the improvements achieved by our intervention but also due to the well-known benefits of physical exercise on various aspects of mental and physical health. These benefits were reinforced by the high adherence of the participants to the program.

Metabolic and physiological changes that are typical during the aging process can lead to significant nutritional deterioration. This deterioration impacts the quality of life of older adults, increasing their dependency on others for daily activities and general care. As a result, it is crucial to address the nutritional status in older adults, emphasizing specific interventions that can help mitigate these effects and support healthier aging [40]. Our study highlights the importance of addressing nutritional status in this vulnerable demographic through targeted interventions, such as a yoga program combined with a Mediterranean diet. While it is true that the effect size may be perceived as small, it is essential to recognize that even modest improvements in nutritional status can have a significant impact on the health and well-being of older adults. In line with our results, there are studies that have observed an association between physical exercise and nutritional status, which can be further enhanced if a specific diet is advised and followed during the exercise program [41]. The improvements achieved in nutritional status demonstrated in this trial contribute to establishing yoga as a useful therapy for this variable, as indicated by other studies [42,43], where the use of yoga also promotes the control of obesity, blood pressure, cholesterol, and fat percentage. Furthermore, the use of the Mediterranean diet throughout the intervention process is of great importance since its beneficial effects on body composition, weight control, functional improvements, and increased quality of life in patients are well known, especially in older adults, as indicated by the studies by Marcos-Pardo et al. [44] and Pérez-Tasigchana et al. [45].

In this particular demographic, it is common to see reduced levels of physical activity, which negatively impact functional areas such as balance, flexibility, and muscle strength. The relationship between yoga practice and balance improvement in older adults is an area of study that has gained significance, highlighting how specific interventions can strengthen these essential physical capabilities [46]. This study analyzes the contribution of various yoga postures to balance and examines the positive influence of the Mediterranean diet in this area, providing a holistic approach that combines exercise and nutrition to optimize the health and safety of this population. Similar results have been found in other studies such as that of Patel et al. [47], a study based on RCTs that evaluated the impact of yoga in the geriatric population and their balance control, concluding that statistically significant results were obtained in balance measured by the Timed Up and Go test between subjects in the yoga therapy group with those who did not receive any intervention. As such, yoga exercises as a complementary therapy are considered to be more therapeutic than traditional exercises and nearly as effective as balance control exercises taught in clinical settings because they require an active engagement of both mind and body. However, other studies [48,49] have reported improvements in balance after yoga interventions, but these results did not reach statistical significance, possibly due to the smaller sample size and differences in yoga methodology and style used. It is important to highlight the beneficial role of the Mediterranean diet in relation to balance, which supports previous findings, such as the study by Tepper et al. [50], who associated the Mediterranean diet with improvements in balance, gait, and risk of falls. Regarding walking, studies such as those by Kelley et al. [51] and Zettergren et al. [25] have shown significant improvements in walking speed after a yoga intervention in older adults, which is consistent with our findings. These improvements in balance and gait suggest a reduction in the risk of falls, as observed in previous research [52,53,54] that employed yoga interventions in older populations, resulting in a decrease in fall occurrences, self-reported symptoms, and a reduction in the fear of falling.

The physical capacity reserve of older people is reduced as they age, and this can affect flexibility [55]. In our findings, it was observed that participants who carried out a combined intervention of yoga and the Mediterranean diet obtained better results in flexibility compared to a control group. Yoga poses involve stretching and weight bearing, which help older adults regain flexibility and increase joint range of motion [56]. Previous studies have compared the effects of yoga on this variable and have found benefits in the group that carried out the intervention, but in other types of samples, such as university students with an average age of 20 years [57] and a study in which only women participated [53]. In addition, yoga protocols different from the one in the present study have been carried out, such as the study by Bucht et al. [36], who also explored the effects of yoga combined with a specific environment, such as a sauna, revealing significant improvements in flexibility, but only of the hamstrings.

Muscular strength is an essential component for maintaining autonomy and quality of life, especially in the later stages of life, where its development and maintenance become a priority [58]. Within mind–body therapies, yoga emerges as one of the best approaches to increase muscle strength along with other functional parameters, yielding positive results regardless of age group [38]. In the present study, both men and women participants who practiced yoga and a Mediterranean diet obtained significant improvements in both grip strength and strength in the lower limbs compared to the control group that did not perform any type of exercise or diet. The mechanisms by which yoga provides beneficial effects on muscle strength remain to be determined, but it is plausible that the benefit is mediated by certain yoga postures, which may have reached an optimal intensity to improve muscle strength and endurance. A previous electromyographic (EMG) analysis in 20 older adults demonstrated that some selected yoga poses, including chair and front warrior, generated considerable moments of force in the knee extensor joint and EMG activity in the quadriceps [59]. Additionally, yoga can help maintain proper posture and spinal alignment, thus exerting beneficial effects on muscle strength and endurance. Previous studies measuring the effects of yoga on muscle strength have yielded conflicting results. Blumenthal et al. [60] showed no changes, while Madanmohan et al. [61] reported significant improvements in handgrip strength as a result of yoga practice. Additionally, several studies [62,63] showed positive associations between the Mediterranean diet and bone and muscle health, promoting increases in muscle strength and adherence to exercise programs.

Among the notable aspects of our research, it is worth highlighting the design we employed: a randomized, controlled, and blinded trial. Likewise, we observed remarkable adherence to the interventions by the participants and had a large sample size, which strengthens the validity of our findings. However, this study also had several limitations. Firstly, only short-term effects were evaluated. Secondly, participants could not be blinded due to the nature of the intervention. Thirdly, despite the observed benefits, it was not possible to determine which results came exclusively from exercise or dietary changes due to the integrated nature of the intervention. In future research, it would be beneficial to implement an experimental design that includes separate groups for each intervention, thus allowing a more precise and detailed evaluation of the causal effects of each component.

## 5. Conclusions

In conclusion, the present study conducted on non-institutionalized older adults demonstrates that a 12-week intervention of yoga supplemented and combined with a Mediterranean diet has beneficial and significant effects on nutritional status, balance, gait, fall risk, flexibility, and muscle strength for the elderly population. Although the evaluation of Cohen’s d value could suggest a small effect in improving nutritional status, it is essential to recognize that even modest changes in this aspect can have significant consequences on the health and well-being of older adults. These results may support the need for public policies that promote physical exercise programs and healthy diets aimed at older adults, as part of disease prevention and health promotion strategies. Comprehensively addressing nutritional status in this demographic group can contribute not only to improving their quality of life but also to reducing the risk of chronic diseases and the associated burden of medical care on the health system.

## Figures and Tables

**Table 1:** Yoga postures from the main part of the training.

Position	Yoga Poses	Description	Illustration	Yoga Poses	Description	Illustration
Standing postures	Virabhadrasana Ⅱ (Warrior Ⅱ)	Similar to Warrior I but with arms stretched out in opposite directions, parallel to the ground.	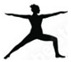	Uttanasana (Standing forward bend)	Entails bending forward from the hips, extending the spine and touching the ground.	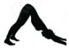
	Vrksasana (Tree Pose)	A balancing pose where one foot is placed on the opposite thigh and hands are raised above the head.	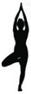	Garudasana (Eagle Pose)	Involves balancing on one foot with the other leg wrapped around the standing leg, and arms intertwined in front.	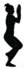
	Naṭarajasana (Dancer Pose)	A challenging balance pose resembling a dancing pose. It strengthens legs and improves balance	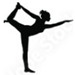	Virabhadrasana I (Warrior Pose)	A lunge pose with one leg forward, arms raised overhead.	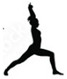
Seated postures	Dasana (Staff Pose)	Sitting with legs stretched out in front and back erect. Strengthens the back muscles and stretches the legs	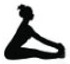	Baddha Konasana (Bound angle Pose)	Feet are joined together with knees dropped to the sides, improving hip flexibility	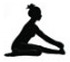
	Parivrtta Janu Sirsasana (Revolved Head-to-Knee Pose)	A twisted variation of Janu Sirsasana enhancing spinal flexibility.	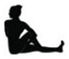	Navasana (Boat Pose)	Balancing on the sit bones with legs and back in a V-shape. Strengthens the core and improves digestion	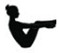
	Gomukhasana (Cockface Pose)	Knees are stacked over each other and hands clasp behind the back. Stretches the hips and shoulders	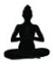			
Kneeling postures	Balasana (Child’s Pose)	Sitting on the heels with forehead on the ground, arms extended.	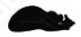	Supta Virasana (Reclining Hero or Heroine Pose)	Kneeling with the back reclined backwards, stretching the thighs and abdominal organs	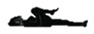
	Ustrasana (Camel Pose)	Kneeling with the torso arched back and hands on the heels	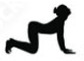	Simhasana (Lion pose)	Kneeling with a powerful exhalation and a roar, beneficial for the vocal cords and respiratory system	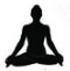
Prone postures	Savasana (Corpse Pose)	Lying flat on the back, used for relaxation and meditation	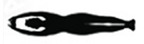	Viparita Karani (Legs-Up-the-Wall Pose)	It is excellent for relaxing the nervous system, reducing swelling in the legs, and easing conditions like anxiety and headaches	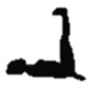
	Halasana (Plow Pose)	An inversion that involves lying on the back and lifting the legs over the head until the toes touch the ground behind.	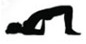	Setubandha Sarvangasana (Shoulder stand)	Often referred to as the queen of poses, this inversion aims to impact the thyroid glands positively, balances hormones, and calms the mind.	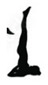
	Jathara Parivrtti Asana (Belly Twist)	This pose enhances digestive health, relieves stress, and can help detoxify the internal organs	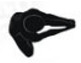			
Supine postures	Bhujangasana (Cow Pose)	A reclining back-bending pose that stretches the chest while strengthening the spine and shoulders	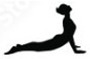	Salabhasana (Locust Pose)	In this pose, the practitioner lies on the belly and lifts the legs and chest off the ground.	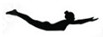

**Table 2:** Initial Characteristics of Study Participants.

		Total (*n* = 116)	Experimental (*n* = 57)	Control (*n* = 59)	*p*-Value
Age		70.03 ± 2.58	70.37 ± 2.61	69.71 ± 2.49	0.471
Sex	Male	35 (30.2)	16 (45.7)	19 (54.3)	0.338
	Female	81 (69.8)	41 (50.6)	40 (49.4)	
Occupational Status	Retired	76 (65.5)	36 (47.7)	40 (52.6)	0.826
	Employed	2 (1.7)	0 (0.00)	2 (100.0)	
	Unemployed	38 (32.8)	19 (50.0)	19 (50.0)	
Marital Status	Single	31 (21.1)	18 (58.1)	13 (41.9)	0.694
	Married	53 (36.1)	27 (50.9)	26 (49.1)	
	Divorced/Separated/Widowed	32 (21.8)	12 (37.5)	20 (62.5)	
Educational Status	No education	23 (19.8)	14 (60.9)	9 (39.1)	0.638
	Primary Education	34 (29.3)	18 (52.9)	16 (47.1)	
	Secondary Education	33 (28.4)	14 (42.4)	19 (57.6)	
	University Education	26 (22.4)	11 (42.3)	15 (57.7)	
Height		1.64 ± 0.13	1.64 ± 0.14	1.66 ± 0.13	0.825
Weight		68.99 ± 13.36	69.00 ± 13.12	68.98 ± 13.68	0.486
BMI		25.12 ± 2.17	25.33 ± 2.45	24.92 ± 1.84	0.298
Adherence to the Mediterranean Diet_MEDAS		6.87 ± 1.02	6.98 ± 1.01	6.76 ± 1.02	0.728
Nutricional Status_MNA		25.32 ± 3.62	25.37 ± 3.47	25.27 ± 3.79	0.337
Balance_Tinetti		9.93 ± 2.61	9.95 ± 2.64	9.92 ± 2.61	0.870
Gait_Tinetti		6.87 ± 2.51	6.67 ± 2.36	7.07 ± 2.66	0.281
Total_Tinetti		16.80 ± 3.90	16.61 ± 3.91	16.98 ± 3.91	0.885
Right Arm_BST		−7.31 ± 9.15	−7.96 ± 9.63	−6.68 ± 8.70	0.098
Left Arm_BST		−6.75 ± 7.82	−5.98 ± 8.33	−7.49 ± 7.28	0.059
Right Leg_CSRT		−9.23 ± 9.62	−11.07 ± 9.13	−7.46 ± 9.82	0.971
Left Leg_CSRT		−13.58 ± 11.61	−15.88 ± 10.23	−11.36 ± 12.49	0.205
Grip Strength		16.58 ± 3.87	16.25 ± 3.82	16.90 ± 3.92	0.843
Lower Body Strength_30s-CST		16.13 ± 2.81	16.04 ± 2.57	16.22 ± 3.05	0.062

Data are expressed as the mean and standard deviation. Qualitative variables are presented as frequency and percentage.

**Table 3:** Effects of yoga on nutritional status, balance, gait, fall risk, flexibility, and muscular strength.

	Post-Intervention		Group			Time			Group × Time		
	EG	CG	F(1.114)	*p*-Value	η^2^	F(1.114)	*p*-Value	η^2^	F(1.114)	*p*-Value	η^2^
Adherence to the Mediterranean Diet_MEDAS	9.16 ± 1.29	6.41 ± 1.23	72.710	0.000	0.389	107.084	0.000	0.484	55.325	0.000	0.327
Nutricional Status_MNA	26.93 ± 3.20	25.07 ± 3.52	2.505	0.116	0.021	11.641	0.001	0.093	19.659	0.000	0.147
Balance_Tinetti	11.12 ± 3.01	10.03 ± 2.35	1.658	0.200	0.014	7.704	0.006	0.063	5.138	0.025	0.043
Gait_Tinetti	7.63 ± 1.96	6.69 ± 2.50	0.432	0.512	0.004	2.869	0.093	0.025	14.651	0.000	0.114
Total_Tinetti	18.75 ± 3.96	16.73 ± 3.55	1.769	0.186	0.015	8.787	0.004	0.072	14.163	0.000	0.111
Right Arm_BST	−3.68 ± 8.53	−7.22 ± 7.98	79.862	0.000	0.412	6.020	0.016	0.050	10.021	0.002	0.081
Left Arm_BST	−3.68 ± 9.23	−9.27 ± 8.13	7.013	0.009	0.058	0.118	0.732	0.001	7.278	0.008	0.060
Right Leg_CSRT	−3.93 ± 4.42	−6.66 ± 8.17	0.117	0.733	0.001	24.338	0.000	0.176	15.548	0.000	0.120
Left Leg_CSRT	−7.93 ± 7.81	−11.93 ± 13.08	0.018	0.894	0.000	27.469	0.000	0.244	36.731	0.000	0.244
Grip Strength	17.67 ± 3.64	16.33 ± 3.22	0.277	0.600	0.002	7.149	0.009	0.059	40.317	0.000	0.261
Lower Body Strength_30s-CST	14.53 ± 2.67	16.71 ± 2.70	6.766	0.011	0.056	4.757	0.031	0.040	18.392	0.000	0.139

Data are presented as the mean and standard deviation. CG = control group, EG = experimental group, F = variance between groups and intra groups, η^2^ = eta square, MNA = Mini Nutritional Assessment, BST = Back Scratch Test, CSRT = Chair Sit-and-Reach Test, 30s-CST = 30s Chair Stand Test.

## Data Availability

The data presented in this study are available on request from the corresponding author. The data are not publicly available because, due to the sensitive nature of the questions asked in this study, participants were assured raw data would remain confidential and would not be shared.
